# Programmable coupled oscillators for synchronized locomotion

**DOI:** 10.1038/s41467-019-11198-6

**Published:** 2019-07-24

**Authors:** Sourav Dutta, Abhinav Parihar, Abhishek Khanna, Jorge Gomez, Wriddhi Chakraborty, Matthew Jerry, Benjamin Grisafe, Arijit Raychowdhury, Suman Datta

**Affiliations:** 1 Department of Electrical Engineering, University of Notre Dame, Notre Dame, IN 46556 USA; 2 School of Electrical and Computer Engineering, Georgia Institute of Technology, Atlanta, GA 30332 USA

**Keywords:** Electrical and electronic engineering, Electronic devices, Electronic properties and materials, Applied mathematics

## Abstract

The striking similarity between biological locomotion gaits and the evolution of phase patterns in coupled oscillatory network can be traced to the role of central pattern generator located in the spinal cord. Bio-inspired robotics aim at harnessing this control approach for generation of rhythmic patterns for synchronized limb movement. Here, we utilize the phenomenon of synchronization and emergent spatiotemporal pattern from the interaction among coupled oscillators to generate a range of locomotion gait patterns. We experimentally demonstrate a central pattern generator network using capacitively coupled Vanadium Dioxide nano-oscillators. The coupled oscillators exhibit stable limit-cycle oscillations and tunable natural frequencies for real-time programmability of phase-pattern. The ultra-compact 1 Transistor-1 Resistor implementation of oscillator and bidirectional capacitive coupling allow small footprint area and low operating power. Compared to biomimetic CMOS based neuron and synapse models, our design simplifies on-chip implementation and real-time tunability by reducing the number of control parameters.

## Abstract (web summary)

Designing alternative paradigms for bio-inspired analog computing that harnesses collective dynamics remains a challenge. Here, the authors exploit the synchronization dynamics of coupled vanadium dioxide-based insulator-to-metal phase-transition nano-oscillators for adaptive locomotion control.

## Introduction

Extremely energy-efficient locomotion control lies at the heart of developing autonomous micro-robots for real-world tasks in an energy-constrained environment and exoskeletons for treating patients with spinal cord injury^1–4^. Enabling synchronized limb movement in such robotic systems with large degrees of freedom, however, presents a technological challenge for traditional model-based centralized control approach where motion patterns are computed globally, and the robot is controlled at the local joint level. This is where neurobiology merges with technology to enable biologically inspired hardware for robotic locomotion control. Pioneering works in neurobiology has now shed light on this close resemblance between phase patterns of coupled oscillators and locomotion gaits through the discovery of the central pattern generator (CPG)^5–7^. CPG involves an ensemble of neural oscillators located in the spinal cord of vertebrates and in ganglions of invertebrates that are intricately involved in producing a multitude of rhythmic patterns like locomotion, breathing, chewing, and so on. CPGs locally give rise to complex rhythmic patterns for locomotion and seamless gait transition while receiving simple input signals from higher regions in the brain. Compared to a traditional centralized control, such a distributed CPG-based decentralized approach (a) reduces the time delays in motor control loop where the rhythmic motion is control by short feedback loops through the spinal cord and (b) reduces the dimensionality of the control signal where instead of specifying the activity of each joint, now the control signal has to only modulate the CPG activity while the functionality of the individual joint is controlled locally by the CPG network itself^1^. Such decentralized CPG-based approach further adds adaptiveness where a robot can modify its gait to continue locomotion despite a damaged limb^8,9^.

In this work, we demonstrate a distributed CPG-based decentralized approach using a network of capacitively coupled insulator-to-metal phase-transition nano-oscillators (IMT-NOs) using vanadium dioxide (VO_2_) that provides a novel nanotechnology fabric for hardware implementation of CPG. Our work relies on an alternative paradigm of biologically inspired analog computing harnessing collective dynamics that involves constructing a network of interacting elements where each component is endowed with dynamical functionality, such as self-oscillations. Harnessing the emergent physical phenomena like synchronization (i.e., frequency locked with constant or bounded phase difference) and the global spatiotemporal dynamics that emerge from the local interactions enables the generation of biologically plausible locomotion gaits. We exploit the synchronization dynamics of coupled VO_2_-based IMT-NOs to experimentally demonstrate a neuromorphic CPG. The coupled IMT-NOs in their synchronized state exhibit stable limit cycle that generates and preserves rhythmic patterns immune to small perturbations. Additionally, the NOs exhibit tunable natural frequencies that enable real-time programmability of phase patterns without requiring to change the coupling strengths between the oscillators in the network. The ultra-compact 1 Transistor-1 R (1T-1R) implementation of IMT-NO with scaling potential down to sub-100 nm and straightforward bidirectional coupling using passive elements like capacitors allow extremely small footprint area and low operating power budget. Compared to the biomimetic neuron and synapse implementations using analog CMOS (complementary metal oxide semiconductor) mentioned earlier, our approach simplifies on-chip implementation and real-time adaptive modulation of generated phase patterns by significantly reducing the number of control parameters. The demonstration of a low-power compact CPG hardware for in situ gait generation and locomotion control provides an important step towards building bio-inspired beyond CMOS hardware with networks of interacting devices and has applications in both autonomous micro-robots operating in energy-constrained environment and wearable exoskeletons for paraplegic patients.

## Results

### CPG-based locomotion control

**Fig. 1:**
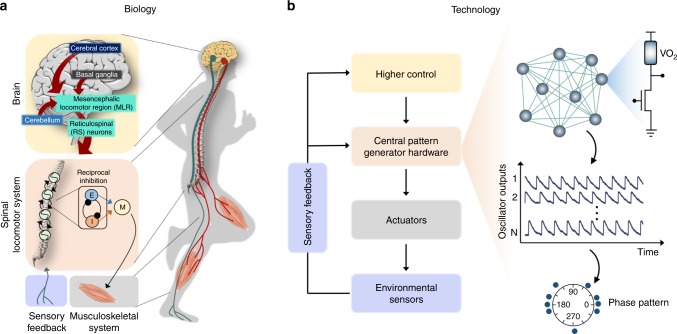
Central pattern generator (CPG)-based locomotion control. **a** Illustration of the signal flow involved during locomotion in a human. **b** Overview of bio-inspired CPG-based robotic locomotion control. The proposed CPG hardware implementation is based on coupled insulator-to-metal phase-transition nano-oscillators (IMT-NOs)

The design of CPG-based locomotion control scheme is heavily inspired by its biological counterpart. The experimental evidence regarding the existence of CPG has been shown in the spinal cord of mammals^5,10–13^ as well as indirectly in humans^7,14–16^. These works also shed light on the fundamental signal flow involved during locomotion^17^. Figure 1a provides a simplified illustration of the signal flow. The simplest spinal CPG architecture that is responsible for the generation of basic locomotion gaits consists of half-center oscillators, HCOs (E and I in Fig. 1a), where each individual neuron fire non-rhythmically when uncoupled, but gives rise to rhythmic pattern when coupled via reciprocal inhibition. HCOs in turn affect the motor neuron that controls locomotion (however, see McCrea and Rybak^10^ for other plausible architectures). CPGs produce complex rhythmic patterns for locomotion and gait transition while receiving simple input signals from higher regions in the brain like the cerebral cortex, cerebellum, basal ganglia, and mesencephalic locomotor region (MLR) in the brainstem^1,17–19^. Figure 1b shows an overview of CPG-based locomotion control scheme for bio-inspired robotics application. The CPG hardware is responsible for rhythmic pattern generation giving rise to inter-limb coordination, while the higher control signals for pattern modulation may come from external sources and through the sensory feedback that detects changes in environment and terrain.

The vast amount of research on CPG-based hardware for locomotion have relied on developing physical implementation of on-chip artificial neurons and synapses with varying degrees of biological realism via means of complex CMOS analog or mixed-signal circuits. For example, Lewis et al.^20,21^ proposed an analog CMOS implementations to mimic a biologically plausible integrate-and-fire neuron. However, such a topology causes significant transient current during the switching of the constituent transistors and hence dynamic power dissipation^22^. Vogelstein et al.^23^ proposed a similar topology for neurons and power hungry digital-to-analog converters to implement synaptic connections. Similar neuromorphic implementation of CPG was been proposed in refs. ^24–26^. The pulse-type hardware-based CPG^27,28^ also suffers from large transistor count and power dissipation. To achieve low-power consumption, sub-threshold circuit techniques were proposed by Lee et al.^29^ and Nakada et al.^30^. However, such implementations still require high transistor count complex analog circuit and face scaling challenges from worsening transistor threshold voltage mismatch. Compared to the biomimetic neuron and synapse implementations using analog CMOS mentioned earlier, our 1T-1R IMT-NOs provide a compact on-chip implementation with low operating power budget. Our work on IMT-NOs with bidirectional coupling is a marked departure from the past oscillator-based CPG implementation by Still et al.^31^, who employed a chain of master-slave CMOS oscillators with unidirectional coupling. Finally, our computation approach leveraging collective dynamics of coupled oscillators provides an energy advantage compared to oscillator-based CPG models implemented on digital microcontroller (e.g., salamander robot developed by Ijspeert et al.^32^) and field-programmable gate array (FPGA)^33–38^.

### IMT-based relaxation oscillator

**Fig. 2:**
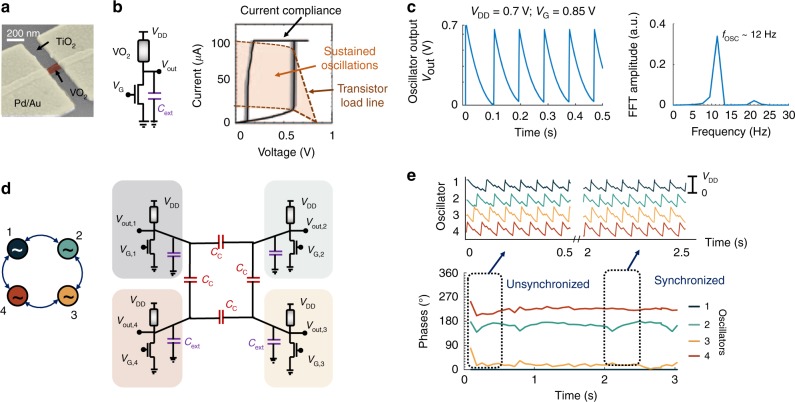
**a** False-colored scanning electron microscope (SEM) micrograph of 200 nm size two-terminal VO_2_ device. **b**, **c** Sustained oscillations are generated by connecting the VO_2_ device in series with a MOSFET (metal–oxide–semiconductor field-effect transistor) in a 1 Transistor-1 Resistor (1T-1R) configuration such that the FET load line intersects the vertical edges of the hysteresis loop of VO_2_ at unstable points. **d** Schematic of a network of capacitively coupled oscillators used for generating phase patterns. **e** Experimentally measured spatiotemporal dynamics of the network. Starting from an initial unsynchronized condition, the oscillators rapidly undergo synchronization and a steady-state spatio-temporal phase pattern of the network emerges as the oscillators remain phase locked

We consider a two-terminal device composed of a phase-transition material VO_2_ as a natural fabric for realizing a relaxation oscillator. It is well known that VO_2_ undergoes electrically induced IMT phase transition^39^ accompanied by orders of magnitude abrupt change in resistivity^40^. The phase transition is hysteretic in nature, meaning that the IMT transition and the MIT transition do not occur at the same voltage. Figure 2a shows a false-colored tilted view scanning electron microscope (SEM) image of the fabricated two-terminal VO_2_ device used in our experiments (see Methods for details) along with the hysteretic direct current current–voltage characteristics of the device (Fig. 2b). The device dimensions used for measurements were $L_{\mathrm{VO}_2}$ = 200 nm and $W_{\mathrm{VO}_2}$ = 100 nm–2 μm. Sustained oscillations can be generated by coupling the two-terminal VO_2_ device in series with a MOSFET (metal–oxide–semiconductor field-effect transistor) in a 1T-1R configuration as shown in Fig. 2b, where the FET load line is tuned to intersect the vertical edges of the hysteresis loop of VO_2_ at unstable points by adjusting the gate voltage, *V*_G_. In other words, bifurcation can be achieved by tuning the load line of the series transistor^40,41^. Figure 2c shows sustained relaxation oscillations obtained at the point of bifurcation with a *V*_DD_ of 0.7 V and *V*_G_ = 1.19 V. We used an external off-chip capacitor, *C*_ext_, to obtain a low frequency of 12 Hz required for practically feasible locomotion. It is to be noted that the metallic conductance of VO_2_ is very high causing the charging to be almost instantaneous followed by a gradual discharge and, in a way, resembles the firing of biological neuron. In fact, one can map the piecewise linear dynamics of the IMT oscillator to that of a piecewise linear approximation of the FitzHugh–Nagumo neuron^42^.

### Synchronization and evolution of phase patterns

Intriguing spatiotemporal dynamics emerge when one considers not a single, but a population of oscillators^43–46^. For example, Cuellar et al.^12^ have reported an electrophysiological evidence of traveling waves produced by neurons within the spinal cord, and Ijspeert et al.^32^ utilized such traveling wave model to demonstrate a swimming salamander robot. The connectivity in such a network can vary from a simple nearest- or few immediate-neighbor couplings to an all-to-all connection. In this work, we focus on the dynamics of one such network where four IMT oscillators are connected in a ring configuration with bidirectional nearest-neighbor coupling. Figure 2d illustrates the schematic of the network where the oscillators are capacitively coupled using off-chip capacitors, *C*_C_ = 200 nF (see Method and Supplementary Fig. 1 for full experimental setup). Using capacitances for coupling (instead of resistance) enable a non-dissipative coupling scheme while ensuring frequency synchronization. Besides modifying the resultant synchronized frequency of the oscillators, the coupling capacitance, *C*_C_, also influences the strength of coupling wherein a high (low) *C*_C_ corresponds to a strong (weak) coupling strength. This is in contrast to resistive coupling wherein a high resistance corresponds to weak coupling. The effect of strong coupling is analogous to inhibitory coupling among spiking neurons, which results in anti-phase oscillations, while weak coupling exerts an excitatory effect resulting in in-phase oscillations. Note that both strong and weak coupling can be achieved using a combination of capacitance and resistance, as shown in Supplementary Fig. 2. Figure 2e shows the spatiotemporal dynamics of the network. Starting from an initial unsynchronized condition, the oscillators rapidly undergo synchronization and a steady-state spatiotemporal phase pattern of the network emerges as the oscillators remain phase locked. Here, we consider the scenario of identical oscillators (by keeping the gate biases *V*_G_ of all oscillator same). The strong capacitive coupling causes the adjacent oscillators to remain out of phase^41^. Hence, the phases of the diagonal oscillators (1, 3) and (2, 4) in the ring configuration cluster together and settle to around 0° and 180°, respectively^47^. Note that we calculate the phases of other oscillators with respect to oscillator 1, which is acts as a reference phase at 0°.

### Modulation and adaptation of phase patterns

While an open-loop CPG-based control scheme is capable of producing rhythmic gait patterns, autonomous dynamic adaptation based on sensory feedback signals is required to cope with varying terrains and avoid obstacles. The feedback signals, arising from various sources like altitude sensor or foot pressure sensor, can directly influence the CPG and modulate its output. In this work, we consider the approach introduced by Barikhan^48^ for adaptive locomotion of hexapod robot. We consider a hard-wired IMT-NO based CPG network where the gait pattern is determined by the phase differences among the oscillators. The phase differences are influenced by the fixed capacitive coupling elements and tunable gate voltages *V*_G_s of the series transistors that dictates the natural frequencies of the individual oscillators^49^. A difference in *V*_G_s lead to a difference in intrinsic frequencies causing phase shifts in the frequency-locked regime. The network is considered to have a nominal gait pattern as shown in Fig. 2e under uniform bias conditions applied to the transistors. The continuous sensory feedback validates the generated gait pattern against the environmental changes and adjusts it by continuously tuning the *V*_G_s. Thus, a modified gait pattern evolves from the nominal pattern through the mutual entrainment of the oscillators in the network and the feedback signals. Such modulation of phase pattern based on feedback signal has also been adapted for obstacle avoidance of robotic fish^50^ and quadruped locomotion on irregular terrain^51^. A detailed design of robotic sensors and related feedback signals is beyond the scope of this work. Hence, we consider a simplified scenario of feedback-driven adaptive gait generation where the present phase pattern has to be modified to a final one more suited for the environment.

**Fig. 3:**
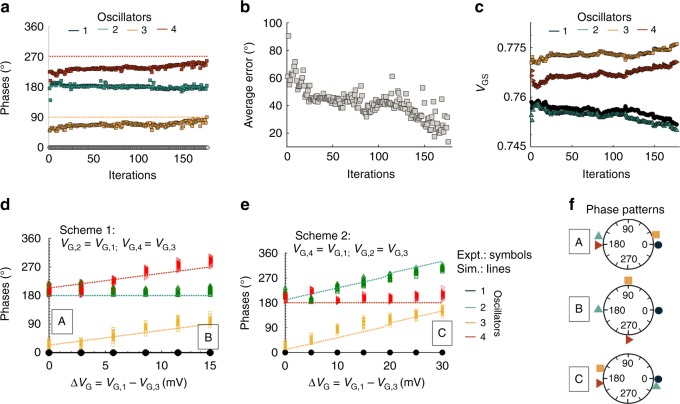
Simulation phase patterns showing the evolution of the insulator-to-metal phase-transition nano-oscillators (IMT-NO) from an initial condition towards the desired phases (shown by dotted lines in the figure). The feedback signal considered here is the error between the current and desired phase pattern. The corresponding *V*_G_’s are modulated based on this feedback error signal (see Method for details). **b**, **c**) show evolution of the error in the phase pattern and *V*_G_’s of the oscillators. **d**, **e** Illustration of two phase-modulation schemes governed by the difference between the gate biases Δ*V*_G_ of oscillator 1 (*V*_G,1_) and oscillator 3 (*V*_G,1_). In scheme 1, *V*_G,2_ = *V*_G,1_ and *V*_G,4_ = *V*_G,3_, while in scheme 2, *V*_G,2_ = *V*_G,3_ and *V*_G,4_ = *V*_G,1_. The experimentally obtained phase differences among the oscillators (shown by symbols) for different Δ*V*_G_s are in excellent agreement with the simulation results (shown by dotted lines). **f** Three representative phase patterns (A, B, and C) obtained from schemes 1 and 2 are also shown

We start by exploring continuous modulation of the phase pattern using a SPICE circuit simulation (see Method for details). Figure 3a shows the simulation result where the IMT-NO phases evolve from an initial condition towards the desired phases (shown by dotted lines in the figure). The feedback signal considered here is the error between the current and desired phase pattern (which in turn maybe proportional to the error in locomotion velocity, joint angle, or body angle). The corresponding *V*_G_s are modulated based on this feedback error signal. Figure 3b, c show evolution of the error in-phase pattern and *V*_G_s of the oscillators.

To further experimentally validate the simulation result of phase-pattern modulation with change in *V*_G_s, we experimentally applied different gate biases *V*_G_s to the oscillators and measured the resultant phase pattern. The experimentally obtained phase differences among the oscillators are shown in Fig. 3d, e using symbols, while that obtained via simulation are shown by dotted lines. Figure 3d, e show two-phase modulation schemes governed by the difference between the gate biases Δ*V*_G_ of oscillator 1 (*V*_G,1_) and oscillator 3 (*V*_G,1_). In scheme 1, we keep the gate voltages of oscillators 2 and 4 to be equal to that of oscillators 1 and 3, respectively (*V*_G,2_ = *V*_G,1_ and *V*_G,4_ = *V*_G,3_). Starting from the condition where all the oscillators are identical and the capacitive coupling forces the adjacent oscillators to remain out of phase, increasing Δ*V*_G_ reduces the phase difference between oscillators 1 and 4 (and similarly between oscillators 2 and 3). In scheme 2, the gate voltages of oscillators 2 and 4 are set to be equal to that of oscillators 3 and 1, respectively (*V*_G,2_ = *V*_G,3_ and *V*_G,4_ = *V*_G,1_). Increasing Δ*V*_G_ reduces the phase difference between oscillators 1 and 2 (and similarly between oscillators 3 and 4).

The experimentally obtained phase differences among the oscillators (shown by symbols in Fig. 3d, e) for different Δ*V*_G_s agree well with the simulation results (shown by dotted lines) using the method previously described. Three representative phase patterns (A, B, and C) obtained from schemes 1 and 2 are also shown in Fig. 3f. It is remarkable that any desired phase pattern can be obtained from the same network by simply changing the input gate biases of the individual oscillators without changing the network topology. This proves to be a useful approach towards the demonstration of the adaptive IMT oscillator-based CPG hardware. Note that the tendency of the oscillatory network to synchronize depends on the width of the frequency distribution of the network compared to the coupling strength^52^. If the spread is large, that is, the oscillators differ a lot in their natural frequencies, compared to the coupling strength, the network becomes unsynchronized as seen for higher values of Δ*V*_G_.

### Phase patterns emulating quadruped locomotion

**Fig. 4:**
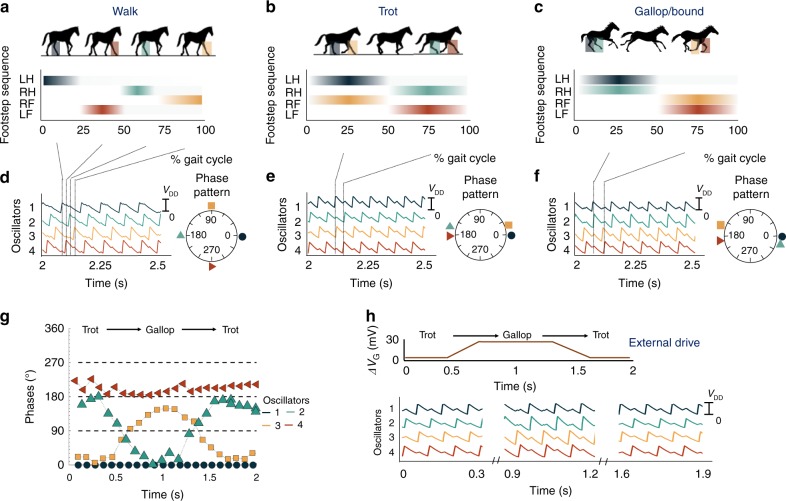
**a**–**c** Typical gait pattern of a quadruped characterized by distinctive phase patterns. Here, LH: left hind; LF: left front; RH: right hind; and RF: right front. **d**–**f** Experimentally obtained spatiotemporal dynamics of the oscillator network and the corresponding generated phase patterns using different biasing schemes of *V*_G_ of the series transistors. Walking gait is realized by utilizing the biasing conditions of scheme 1 (*V*_G,2_ = *V*_G,1_, *V*_G,4_ = *V*_G,3_) and setting Δ*V*_G_ (=*V*_G,1_ − *V*_G,3_) = 15 mV. Trotting gait is realized by applying *V*_G_ = 1.19 V to all the series transistor gates. Galloping or bounding gait is realized by scheme 2 bias scheme with Δ*V*_G_ = 30 mV. **g**, **h** Measured dynamic modulation of the spatiotemporal pattern using change in Δ*V*_G_ allowing seamless adaptive gait transition between a trot and a gallop. A smooth transition in the phase pattern between a walk and a trot upon applying a Δ*V*_G_ = 30 mV using scheme 2

Next, we explore the close resemblance between the phase patterns obtained from our IMT-NO-based CPG network and three quintessential quadruped animal locomotion gaits—walk, trot, and gallop/bound. An ambling horse lifts each foot in turn in a sequence: left hind (LH)–left front (LF)–right hind (RH)–right front (RF) with 90° phase difference between each leg (see Fig. 4a). The trot gait is characterized by the diagonal limbs (LH, RF) and (LF, RH) moving in-phase with 180° phase difference between the two pairs as shown in Fig. 4b. The gallop/bound gait has the characteristics of the front legs (LF, RF) moving together (or with a slight phase difference between the two) and 180° out of phase with the hind legs (LH, RH) as shown in Fig. 4c. The phase pattern corresponding to the walking gait is realized by utilizing the biasing conditions of scheme 1 (*V*_G,2_ = V_G,1_, *V*_G,4_ = *V*_G,3_) and setting Δ*V*_G_ (=*V*_G,1_ − *V*_G,3_) = 15 mV. Figure 4d shows the experimentally obtained spatiotemporal dynamics of the coupled oscillator network and the corresponding generated phase pattern. The phase pattern for trot is evoked by the natural dynamics of the coupled oscillator network in the ring configuration without any asymmetric biasing scheme of the oscillator gate voltages. To demonstrate trot gait, we apply *V*_G_ = 1.19 V to all the series transistor gates. The time-domain voltage waveforms for all the oscillators and the generated phase pattern is shown in Fig. 4e. The gallop/bound gait is realized by scheme 2 biasing with Δ*V*_G_ = 30 mV. Figure 4f shows the generated spatiotemporal pattern corresponding to galloping gait.

We additionally explore the possibility of seamless gait transition. Analogous to the descending signal from the MLR of the brain, we utilize the gate signal modulation Δ*V*_G_ in our IMT-NO-based CPG that acts as the external stimulus and gives rise to spontaneous gait transition. Figure 4g illustrates the experimentally obtained smooth transition in the phase pattern between a trot and a gallop upon applying a Δ*V*_G_ = 30 mV using scheme 2. The measured output voltage signals from the oscillators are also shown in Fig. 4h showing a seamless evolution of the spatiotemporal pattern. Note that all the oscillators remain phase locked during the gait transition with minimal phase error due to the strong inter-oscillator coupling.

**Fig. 5:**
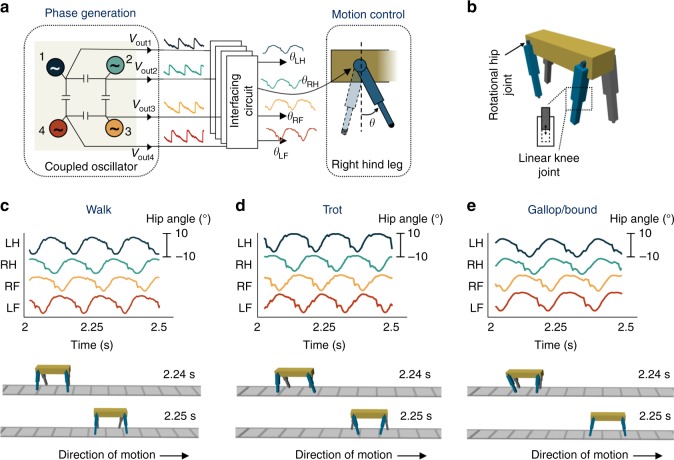
Robotic simulation illustrating insulator-to-metal phase-transition (IMT) oscillator-based central pattern generator (CPG) hardware for real-time quadruped locomotion. The simulation setup is shown in **a**, **b**. The experimentally measured voltage outputs *v*_*i*_(*t*) from the oscillator network are transformed into rotational hip joint angles of the quadruped using a simple transformation ${\theta}_{{\mathrm{hip}},i} = {f}\left( {v_{i}\left( t \right)} \right)$, where *f* can be a linear/non-linear function depending on the target angle waveform. **c**–**e** Temporal evolution of the hip angles of the quadruped obtained from the IMT oscillator-based CPG for the three different gaits and the snapshots of locomotion obtained from the robotic simulation

We characterized the IMT-NO-based CPG hardware for quadruped locomotion using a robotic simulation in Simscape^53^ (see Methods for simulation details). The simulation setup is shown in Fig. 5a, b. The rhythmic voltage outputs *ν*_*i*_(*t*) measured from the oscillators in the network are transformed into rotational hip joint angles of the quadruped using a simple transformation ${\theta }_{{\mathrm{hip}},i} = {f}\left( {v_{i}\left( {t} \right)} \right)$, where *f* can be a linear/non-linear function depending on the target angle waveform. Additional low-pass filtering may be applied to eliminate sharp voltage spikes due to the abrupt IMT. For interfacing with the servomotor of an actual robot, one can design an interfacing circuit that is beyond the scope of this work. Figure 5c–e shows the temporal evolution of the hip angles of the quadruped obtained from the IMT oscillator-based CPG for the three different gaits and the snapshots of locomotion obtained from the robotic simulation. Note that the hip angles reflect the same phase patterns as that obtained in Fig. 3d–f.

## Discussion

Overall, the IMT-NOs fulfill the key requirements for design and implementation of a CPG hardware. The coupled IMT-NOs exhibit stable limit-cycle immune to small perturbations from noise or device variability. The frequency of the individual oscillators can be modulated by tuning the gate voltage of the respective series transistors that, in turn, allows modulation of phase pattern in a coupled network. The amplitude of the oscillation can be varied by varying the applied *V*_DD_. However, the duty factor (here defined as the ratio of the charging time to the total time period) relies on the charging and discharging time of the relaxation oscillator. As shown in the SPICE circuit model in Supplementary Fig. 3, the charging time replies on the metallic resistance of VO_2_, while the discharging time is dictated by the series transistor. Generally, the metallic conductance of VO_2_ is very high causing the charging to be almost instantaneous followed by a gradual discharge. However, by using an additional transistor in the charging path, we can separately modulate the charging and discharging time constants, thus modulating the duty cycle of the oscillations. In summary, the IMT-NOs provide a power efficient compact hardware platform for implementing CPG for adaptive and synchronized locomotion.

A few practical challenges need to be addressed before implementation of a real robot. First, harnessing the phase dynamics of coupled oscillatory networks for CPG-based locomotion requires that the steady-state solution of the network reflects the correct phase ordering of the self-oscillating nodes (including lead or lag). This may pose a problem when one considers that the synchronization of complex dynamical networks is often characterized by multiple cluster synchronization and existence of multiple phase patterns (or limit cycles) based on the topology of the network^54,55^. By applying symmetry arguments, we found the existence of six possible limit cycles (considering not only the ordering of nodes but also a lead or lag in-phase difference) in our four-oscillator network connected in a ring configuration. This may result in a random distribution of phase patterns generated by the CPG for a cold start (random initial condition). However, one can achieve a deterministic phase pattern for locomotion by using a start-up circuit to orchestrate a programmed start. This will force the oscillators to settle correctly to the desired phase pattern as determined by the initial programmed node voltages (see Supplementary Note 1 for details). Second, the electronic phase transition in VO_2_ is often associated with stochasticity. Recent work on characterizing and modeling the stochasticity revealed that the IMT phase transition is characterized by an avalanche effect where initially a few metallic domains within the otherwise insulating matrix are formed under the influence of an external electric field that, in turn, triggers the rapid emergence of clusters of metallic filaments through a positive feedback^56^. The stochasticity manifests itself as a Gaussian distribution of the IMT triggering voltage *V*_IMT_. Since the IMT determines the termination of the discharge cycle of the relaxation IMT oscillator, the *V*_IMT_ variation in turn causes a fluctuation of the oscillation period. Recently, a rigorous mathematical treatment has shed light on the fact that the discharge phase of the relaxation IMT oscillation can be described by an Ornstein–Uhlenbeck process with a fluctuating boundary condition^42^. Overall, such a cycle-to-cycle jitter can cause sudden phase-breaking event and catastrophic loss of synchronization in the coupled oscillatory network. However, since the synchronization is a strong function of the strength of the coupling, an increase in the coupling strength (proportional to coupling capacitance *C*_C_ in our network) results in a significant reduction of the phase error (see Supplementary Note 2 for details) to acceptable level. Lastly, the IMT-NOs display stable self-sustained oscillation with a range of operating temperature^57^. The upper bound of the operating temperature is set by the insulator-to-metallic phase-transition temperature (*T*_IMT_). *T*_IMT_ is a material property that can be tuned using strain engineering^58,59^ or doping, and has been shown to be around 95 °C with Ge doping^60^. The lower bound is set by *V*_DD_ such that *V*_DD_ is greater than the IMT phase transition triggering voltage (*V*_IMT_). *V*_IMT_ shows an inverse dependence on the operating temperature^61^ and as such a large temperature drop can cause the *V*_IMT_ to become greater than the *V*_DD_, making the device get stuck in the insulating state and the self-oscillations will stop. Shukla et al.^61^ have reported measurements of stable oscillations at near room temperature (18 °C).

In conclusion, we experimentally demonstrate a network of bidirectional capacitively coupled IMT-NOs as an efficient hardware platform that can potentially implement CPG for adaptive and synchronized locomotion. Compared to the previous works on pairwise-coupled IMT oscillators^49,61^, this is the first experimental demonstration of a programmable network of IMT-NOs that allows real-time seamless modulation of the emergent phase patterns to enable adaptive synchronized locomotion. While this work focused on the dynamics of a smaller network with four IMT oscillators connected in a ring configuration, it will be plausible to extend this research towards demonstration of a larger network of IMT-NOs to reproduce traveling wave phenomenon of CPG circuit^12,32^ and explore phase resetting^62^ between multiple groups of such coupled IMT-NOs. The ability for voltage-controlled frequency modulation of individual IMT-NOs is consistent with CPGs in the cat spinal cord, which can exhibit a change in frequency from scratching (up to 5 Hz) to locomotion (around 1 Hz)^63^ and opens the door for future exploration of further frequency tunability over a wider range from 12 Hz down to 5 or 1 Hz. The coupled oscillator-based CPG exhibits the ability to generate multiple phase patterns that mimics the basic locomotion gaits of a quadruped (walk, trot, and gallop/bound), supports seamless gait transition and displays robustness to device variability and noise. The dimensional scaling potential of the constituent VO_2_ devices while retaining their electronic phase-transition property allows a significant reduction of the operating power down to 8 μW, as demonstrated in the 200 nm × 100 nm × 10 nm size VO_2_ devices, presented in this work. Interfacing the oscillator output to servomotors would require a voltage amplification to around 5 V. Applying a *V*_DD_ = 5 V (as opposed to the currently used value of 0.7 V) will increase the amplitude of the oscillator’s output signal; however, this would also increase the energy dissipation by 7×. Alternatively, one can design a low-power interfacing circuit that allows voltage amplification with lower energy overhead. However, the design and energy estimation of such an interfacing circuit lies beyond the scope of this paper. The low dynamic power dissipated by the individual IMT-NOs combined with the passive bidirectional capacitive coupling element provides a fundamental advantage over CMOS based ASIC implementation in terms of energy. Finally, the compact and biomimetic 1T-1R implementation of IMT-NO offers markedly reduced circuit complexity in terms of device count. Compared to the biomimetic approach of CPG design relying on complex neuronal and synaptic circuitry, our approach simplifies on-chip implementation and real-time tuning capability by significantly reducing the number of control parameters. Table 1 summarizes the comparison of the IMT-NO-based CPG hardware with various CMOS-based CPG implementations. Such an adaptive, low-power, and area-efficient CPG hardware for in situ gait generation and locomotion control provides an important step towards building bio-inspired beyond CMOS hardware with networks of interacting devices, and can find applications in autonomous micro-robots operating in energy-constrained environment, as well as wearable exoskeletons for paraplegic patients.

**Table 1:** Comparison of IMT oscillator-based CPG with various CMOS ASIC implementations highlighting an advantage in terms of power and circuit complexity (transistor count)

	Vogelstein et al.^23^	Yang et al.^26^	Saito et al.^28^	Lee et al.^29^	Nakada et al.^30^	Still et al. ^31^	This work (IMT-NO)
Technology	500 nm	350 nm	350 nm	250 nm		1.2 *μ*m	$L_{\mathrm{VO}_2}$ = 100 nm
Abstraction level	Neuron and synapse	Neuron and synapse	Neuron and synapse	Neuron and synapse	Neuron and synapse	Coupled oscillator	Coupled oscillator
Neuron model	Integrate-and-fire	Matsuoka	Pulse-type hardware	Hindmarsh– Rose	Amari Hopfield	–	–
Coupling model	Programmable DAC as synapse	Constant and inhibitory synapse	Inhibitory synapse	Chemical synapse	Excitatory and inhibitory synapse	Transmission gate as coupling circuit	Capacitor as coupling element
Coupling type	Unidirectional	–	Unidirectional	Unidirectional	Unidirectional	Unidirectional	Bidirectional
Transistor count	–	1296	76	–	244	72	4
Power	8.3 mW	60 μW	138 μW	4.8 mW	50 μW	–	32 μW
Frequency	–	0.5–2 Hz	2 Hz	–	1–1.4 Hz	0.5–2 Hz	12 Hz
Energy/cycle	–	30 μJ/cycle	69 μJ/cycle	–	35 μJ/cycle	–	2.67 μJ/cycle

*IMT* insulator-to-metal phase transition, CPG central pattern generator, *CMOS* complementary metal oxide semiconductor, *ASIC* application specific integrated circuit, *DAC* digital-to-analog converters

## Methods

### Samples

Two-terminal IMT devices as shown in Fig. 2a are fabricated on a 10 nm VO_2_ thin film synthesized using reactive oxide molecular beam epitaxy on (001) TiO_2_ substrate using the Veeco Gen10 system^64^. Planar devices of length 100–200 nm are defined by standard electron beam lithography, followed by Pd (20 nm)/Au (60 nm) contacts deposited using electron beam evaporation and liftoff. The device widths are then defined using CF_4-_ based dry etch and ranged from 100 nm to 2 μm.

### Experimental setup

Figure 2b shows the schematic for a single IMT-NO, which is realized by externally connecting the two-terminal VO_2_ device with an *n*-channel MOSFET (ALD1103). The nominal gate voltage *V*_G_ of the series transistor is 1.19 V, unless otherwise mentioned, and a *V*_DD_ of 0.7 V is used in the experiment. All the experimental results reported in this manuscript were performed using a Keithley 4200 source measure unit and an Agilent 81150A pulse generator to generate voltage pulses and measure current. The oscillatory voltage outputs are measured from the terminal *V*_out_ using an Agilent DSO9104A oscilloscope. Supplementary Figure. S1a shows the schematic of the full experimental setup with four IMT-NOs. Supplementary Figure S1b shows the SEM of the fabricated four IMT-NOs measured using the Keithley 4200-SCS probe station. Discrete off-chip components are used for the remaining circuit, including series transistors, external capacitors *C*_ext_, and coupling capacitors *C*_C_ as shown in Fig. S1c. We used *C*_ext_ = 1 μF for each oscillator to achieve a low frequency of 12 Hz and coupling capacitor *C*_C_ = 200 nF.

### Simulation

SPICE circuit simulation of VO_2_ oscillator allows exploring the synchronization dynamics of complex oscillator network^65^. We performed SPICE circuit simulation of the IMT-NO-based CPG network to explore the possibility for continuous phase-pattern modulation. The circuit for a single IMT-NO is shown in Supplementary Fig. 3. The VO_2_ model is based on a previous work^65^. We used an insulating and metallic resistance of 28 and 3.628 kΩ, respectively. The intrinsic time for IMT phase change was assumed to be 75 fs. Each simulation was run for a simulation time of 2 s with a time-step of 100 ns.

### Data post-processing and algorithm for modulating phase pattern

The oscillatory voltage waveforms recorded experimentally using the oscilloscope was processed later in MATLAB to calculate the phase differences among the oscillators and the generated phase pattern. FFT was performed to ensure the oscillators remained in synchronization. The phase difference between two oscillators was calculated as the time difference between the minima points of the discharging phase of the relaxation oscillators divided by the time period (see Supplementary Fig. 4). The phase difference was averaged over 10 experimental runs and 100 oscillations per run to include cycle-to-cycle stochastic fluctuations. The continuous-time phase modulation was not performed experimentally in real time due to overhead imposed by external instrumentation and software-based signal processing.

For modulating phase pattern by continuously tuning *V*_G_s of the transistors as shown in Fig. 3a–c, we perform SPICE circuit simulation. We start from a random initial value of the *V*_G1–4_ that gives rise to random initial phase of the oscillators and iteratively tune the V_G_s till the oscillator phases match the desired phases. In each iteration *i*, we first run the SPICE simulation for a simulation time of 2 s and calculate the oscillator phases with respect to oscillator 1, hereby referred to as [*ϕ*_12_ *ϕ*_13_ *ϕ*_14_]. Then, we calculate the error between the present and the desired phases [$\widetilde {\phi _{12}}$ $\widetilde {\phi _{13}}$ $\widetilde {\phi _{14}}$]:

$$\left[ {\begin{array}{*{20}{c}} {{\it{\epsilon }}_{12}} \\ {{\it{\epsilon }}_{14}} \end{array}} \right] = \left[ {\begin{array}{*{20}{c}} {\phi _{12} - \widetilde {\phi _{12}}} \\ {\left. {\phi _{14} - \widetilde {\phi _{14}}}\right] } \end{array}} \right].$$

Based on this, we calculate the change in gate voltages,

$$\left[ {\begin{array}{*{20}{c}} {{\mathrm{\Delta }}V_{{\mathrm{G}},2}} \\ {{\mathrm{\Delta }}V_{{\mathrm{G}},4}} \end{array}} \right] = \alpha \left[ {\begin{array}{*{20}{c}} {\frac{{\delta {\it{\epsilon }}_{12}^2}}{{\delta V_{{\mathrm{G}},2}}}} \\ {\frac{{\delta {\it{\epsilon }}_{14}^2}}{{\delta V_{\mathrm{G},4}}}} \end{array}} \right].$$

We used an adaptive learning rate *α* for convergence. The corresponding *V*_G_s for the *i* + 1 iteration are updated according to the following equation:

$$\left[ {\begin{array}{*{20}{c}} {\begin{array}{*{20}{c}} {V_{\mathrm{G},1}} \\ {V_{\mathrm{G},2}} \\ {V_{\mathrm{G},3}} \end{array}} \\ {V_{\mathrm{G},4}} \end{array}} \right]_{i + 1} = \left[ {\begin{array}{*{20}{c}} {\begin{array}{*{20}{c}} {V_{\mathrm{G},1}} \\ {V_{\mathrm{G},2}} \\ {V_{\mathrm{G},3}} \end{array}} \\ {V_{\mathrm{G},4}} \end{array}} \right]_i + \left[ {\begin{array}{*{20}{c}} {\begin{array}{*{20}{c}} {{\mathrm{\Delta }}V_{\mathrm{G},2} + {\mathrm{\Delta }}V_{\mathrm{G},4}} \\ { - {\mathrm{\Delta }}V_{\mathrm{G},2} + {\mathrm{\Delta }}V_{\mathrm{G},4}} \\ { - {\mathrm{\Delta }}V_{\mathrm{G},2} - {\mathrm{\Delta }}V_{\mathrm{G},4}} \end{array}} \\ {\Delta V_{\mathrm{G},2} - \Delta V_{\mathrm{G},4}} \end{array}} \right]_i.$$

### Robotic simulation

Robotic dynamic simulations are performed using Simscape^53,66^ within the Simulink^®^ environment. The physical model has a rigid body with dimensions of 1.5 cm × 0.3 cm × 0.45 cm and a mass of 20 g to which each of the four legs are connected using revolute hip joints. Each upper and lower leg is 0.5 cm long and 6 g connected using a prismatic knee joint. The total mass is equal to 68 g. We use rotational hip joints whose angles are derived from the voltage outputs of the four oscillators using a simple transformation and low-pass filtering to eliminate sharp voltage spikes due to the abrupt IMT. The knee joints are chosen to be linear similar to ref. ^67^, which enables leg retraction and extension during the swing and stance mode of locomotion, respectively. The target leg retraction and extension lengths are obtained from the voltage outputs of the four oscillators with amplitude scaling and 90° phase shift. The contact forces between the leg and ground include normal force, frictional force, and an additional contact law as a linear spring-damper force. The quadruped motion is restricted to the sagittal plane for simplicity. Adaptive time-step Dormand–Prince method is used for numerical integration with an average time-step of 2 ms. The total simulation time is 4 s. No feedback sensors were incorporated in the robotic simulation.

## Supplementary information

Supplementary Information

## Data Availability

The data that support the findings of this study are available within the article and the associated Supplementary material. Any other data are available from the corresponding author upon request.
