# Supplementary material for: Programmable coupled oscillators for synchronized locomotion
Source: Nat Commun. 2019 Jul 24;10:3299. doi: 10.1038/s41467-019-11198-6 (PMC6656780; doi:10.1038/s41467-019-11198-6)
Supplement: Supplementary file 1 — Supplementary Information [file 41467_2019_11198_MOESM1_ESM.pdf]

Supplementary Information

Programmable Coupled Oscillators for Synchronized Locomotion

Sourav Dutta[1*], Abhinav Parihar[2], Abhishek Khanna[1], Jorge Gomez[1], Wriddhi Chakraborty[1], Matthew Jerry[1], Benjamin Grisafe[1], Arijit Raychowdhury[2] and Suman Datta[1]

[1]Department of Electrical Engineering, University of Notre Dame, Notre Dame, IN 46556, USA
[2]School of Electrical and Computer Engineering, Georgia Institute of Technology, Atlanta, GA 30332, USA

* Correspondence to sdutta4@nd.edu

Supplementary Figure 1: Experimental Setup

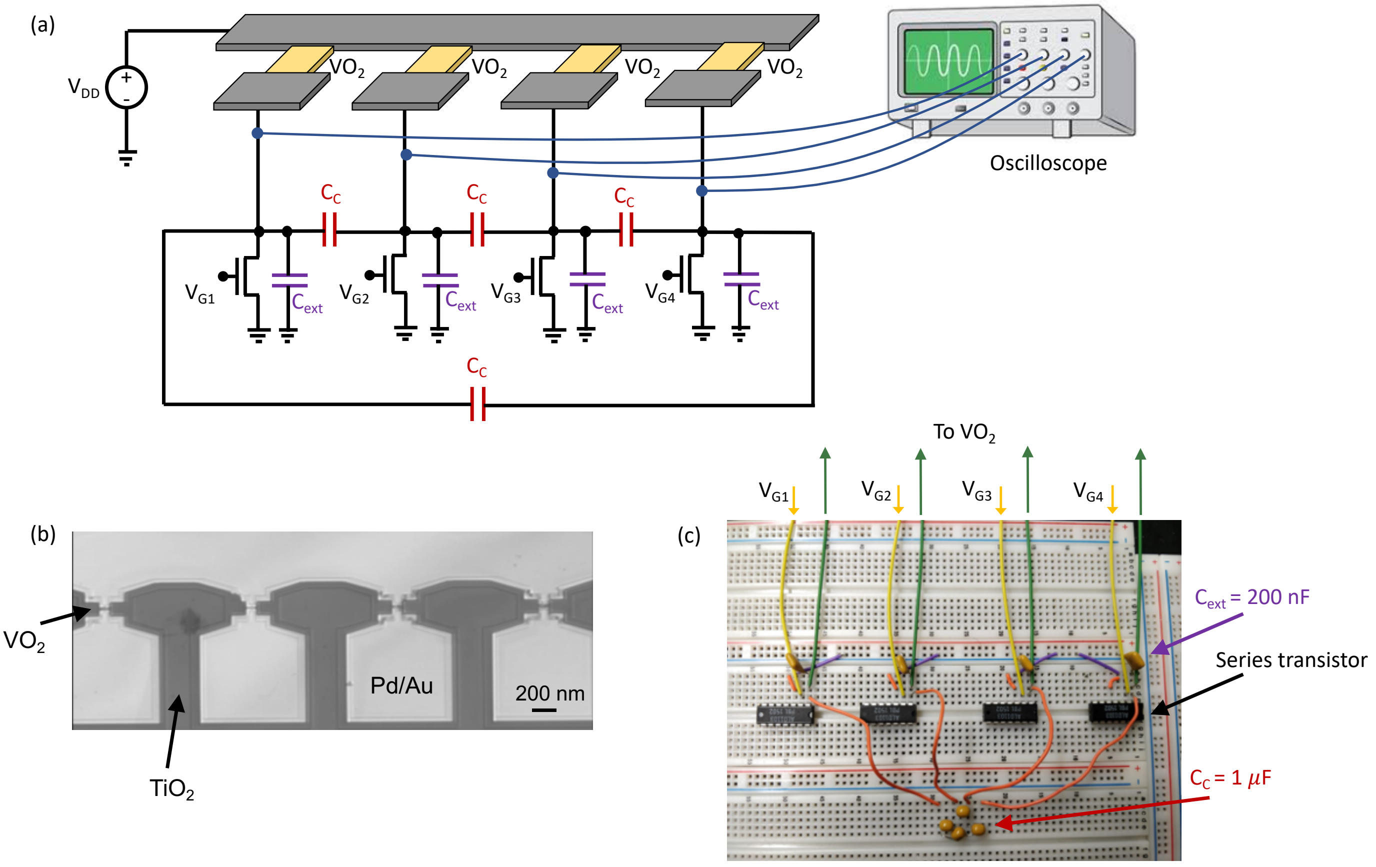

Supplementary Figure 1. Schematic of the complete experimental setup with four IMT-NOs on a chip measured using Keithley 4200-SCS probe station and the remaining circuit constructed using discrete off-chip circuit elements including series transistors, external capacitors C_{ext} and coupling capacitors C_C.

Supplementary Figure 2: Impact of Coupling Strength on Phase Difference

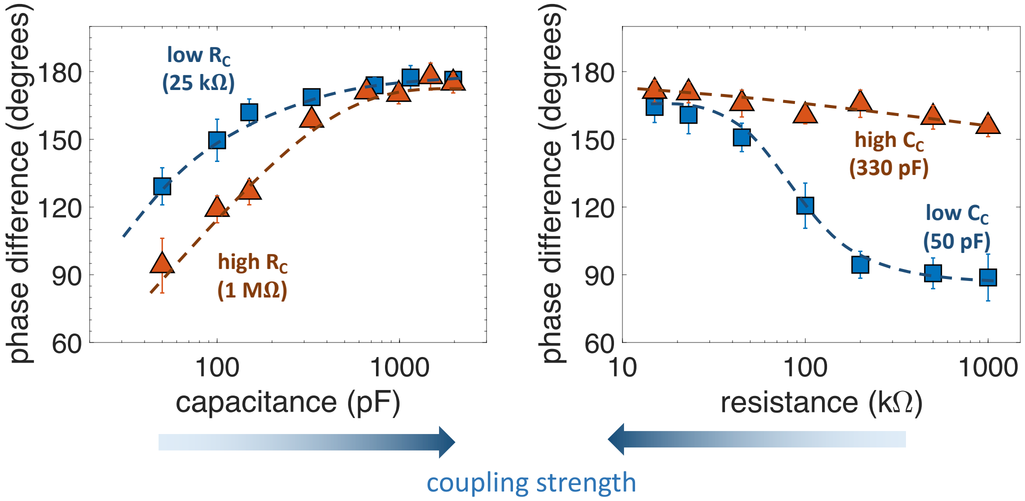

Supplementary Figure 2. Measured phase difference between two pair-wise coupled oscillators as a function of the coupling strength (capacitance and resistance). High capacitive coupling corresponds to a strong coupling strength while a high resistive coupling corresponds to weak coupling. The effect of strong coupling is analogous to inhibitory coupling leading to anti-phase oscillations while weak coupling induces an excitatory effect resulting in in-phase oscillations.

Supplementary Figure 3: SPICE Circuit Model

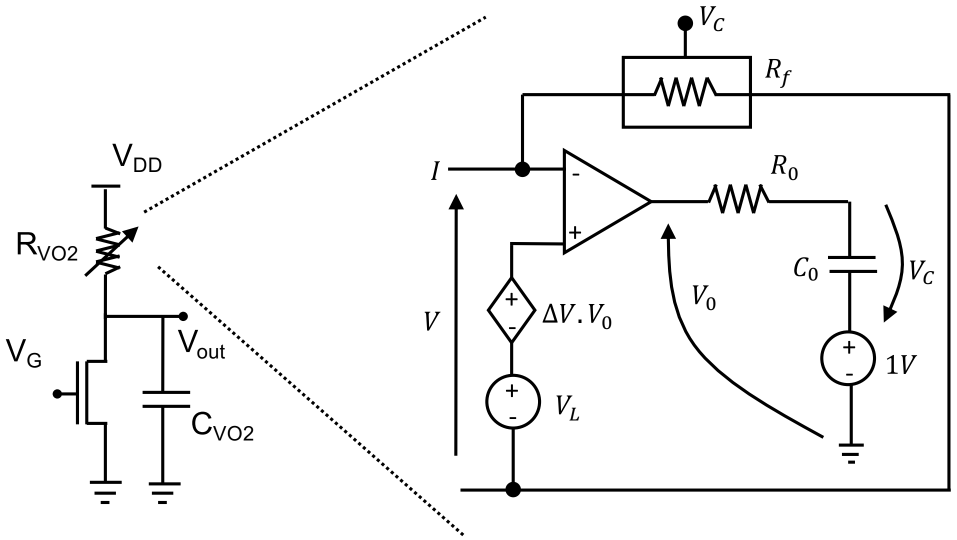

Supplementary Figure 3. SPICE implementation of the circuit model for simulating IMT-NO. The VO_2 model is based on a previous work[1].

Supplementary Figure 4: Phase Difference Between Oscillators

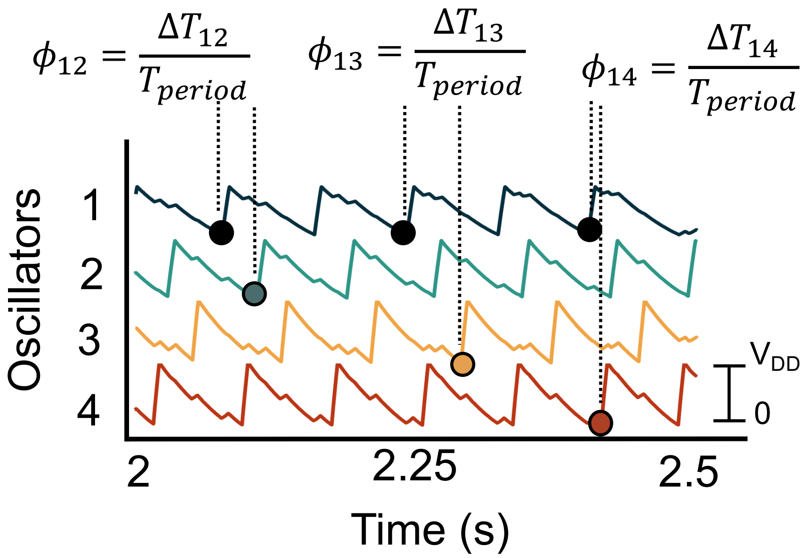

Supplementary Figure 4. The phase-difference between two oscillators was calculated as the time difference between the minima points of the discharging phase of the relaxation oscillators divided by the time period.

Supplementary Figure 5: Initialization of phase pattern

(a)

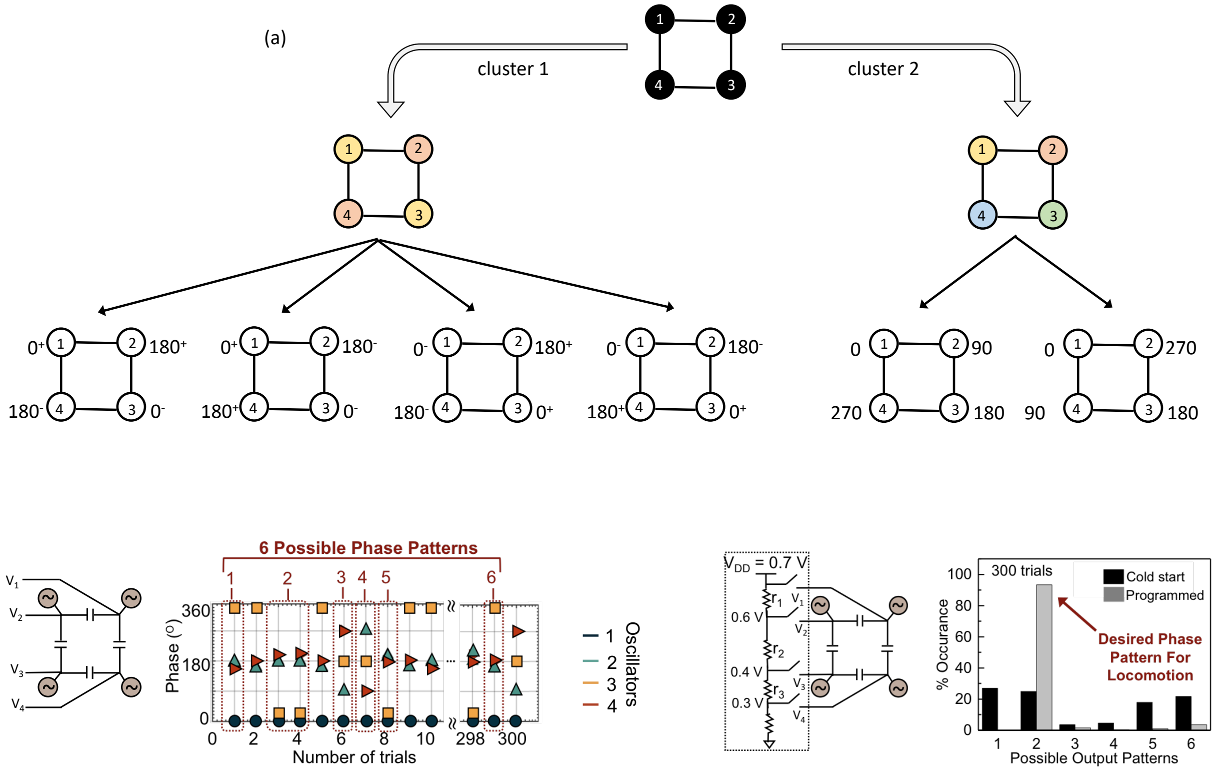

(b) (c)

Supplementary Figure 5. (a) The ring configuration of four-coupled oscillator network exhibits multiple limit cycles considering both ordering of nodes and a lead or lag in phase difference. Note, the '+' sign denotes lead and '-' denotes lag. (b) A random distribution of phase-patterns is generated in case of a cold start (random initial condition). (b) Programmed start with a start-up circuit forces the oscillators to settle to the desired phase-pattern for locomotion as seen in (c).

Supplementary Figure 6. Robustness to variation and noise

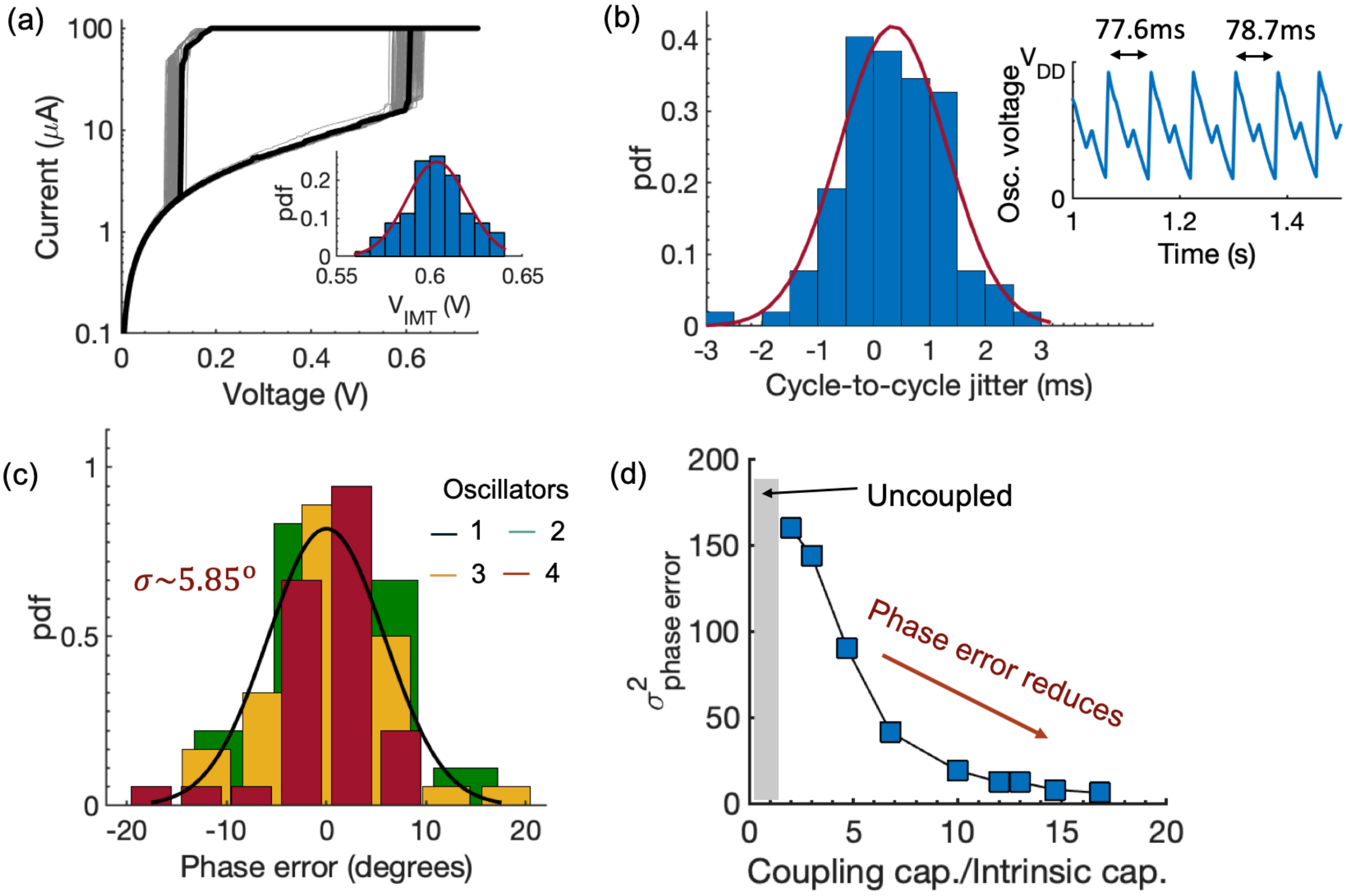

Supplementary Figure 6. (a) DC cycle-to-cycle variation of V_{IMT} causes fluctuation of oscillation period resulting in (b) cycle-to-cycle jitter and (c) phase error. (d) Increasing the coupling strength (proportional to coupling capacitance C_C) in the oscillator network significantly reduces phase error.

Supplementary Note 1. Initialization of phase pattern

Considering our four-oscillator network connected in a ring configuration with bi-directional coupling, simple symmetry arguments can be applied to analyze the possible limit cycles in such networks. The ring configuration of capacitively coupled IMT oscillators exhibits primarily two types of clustering: (i) oscillators (1, 3) are in-phase and 180° out-of-phase with (2, 4), and (ii) all oscillators remain at a phase-difference of 90° with respect to each other. In case of clustering (i), due to the presence of noise and oscillator variations, the oscillators do not exactly settle to 0° or 180°. Thus, oscillators 1 and 3 settle around 0°, but with either a lead (denoted here by 0^+) or lag (0^-). Similarly, oscillators 2 and 4 settle around 180°, but with either a lead (180^+) or lag (180^-). Overall, one can obtain six possible limit cycles as shown in Supplementary Figure 5(a). This results in a random distribution of the generated phase-patterns from a cold start (random initial condition) as shown in Supplementary Figure 5(b) for 300 trials. To achieve a deterministic phase-pattern for locomotion, we use a start-up circuit (Supplementary Figure 5(c)) to enable a programmed start. This forces the oscillators to settle to the desired phase-pattern (Supplementary Figure 5(c)) as programmed by the initial node voltages.

Supplementary Note 2. Robustness to variation and noise

VO_2 exhibits stochastic IMT switching due to fluctuations in the nucleation of the metallic filament[2]. Supplementary Figure 6(a) shows the DC cycle-to-cycle variation of the IMT triggering voltage, V_{IMT}, in a single VO_2 device (L = 200nm). Since the RC discharge time dominates the oscillation period, even a small V_{IMT} variation results in a large fluctuation of the oscillation period. Supplementary Figure 6(b) shows the measured cycle-to-cycle jitter of an IMT oscillator. Supplementary Figure 6(c) shows the measured phase-error resulting from oscillator jitter. The synchronization ability of an oscillator network is directly related to the strength of the coupling elements. An increase in the coupling strength (proportional to coupling capacitance C_C) results in a significant reduction of the phase error as shown experimentally in Supplementary Figure 6(d).

Supplementary References

1. Maffezzoni, P., Daniel, L., Shukla, N., Datta, S. & Raychowdhury, A. Modeling and Simulation of Vanadium Dioxide Relaxation Oscillators. *IEEE Trans. Circuits Syst. I Regul. Pap.* **62**, 2207–2215 (2015).
2. Jerry, M., Ni, K., Parihar, A., Raychowdhury, A. & Datta, S. Stochastic Insulator-to-Metal Phase Transition-Based True Random Number Generator. *IEEE Electron Device Lett.* (2018). doi:10.1109/LED.2017.2771812
